# Towards a ‘people and nature’ paradigm for biodiversity and infectious disease

**DOI:** 10.1098/rstb.2023.0259

**Published:** 2025-01-09

**Authors:** Rory Gibb, David W. Redding, Sagan Friant, Kate E. Jones

**Affiliations:** ^1^ Centre for Biodiversity and Environment Research, Department of Genetics, Evolution and Environment,, University College London, London WC1E 6BT, UK; ^2^ Science Department, Natural History Museum, London SW7 5BD, UK; ^3^ Department of Anthropology, Pennsylvania State University, University Park, PA 16802, USA; ^4^ Huck Institutes of the Life Sciences, Pennsylvania State University, University Park, PA 16802, USA

**Keywords:** disease ecology, infectious disease, biodiversity, socio-ecological systems, epidemiology, global change

## Abstract

Zoonotic and vector-borne infectious diseases are among the most direct human health consequences of biodiversity change. The COVID-19 pandemic increased health policymakers’ attention on the links between ecological degradation and disease, and sparked discussions around nature-based interventions to mitigate zoonotic emergence and epidemics. Yet, although disease ecology provides an increasingly granular knowledge of wildlife disease in changing ecosystems, we still have a poor understanding of the net consequences for human disease. Here, we argue that a renewed focus on wildlife-borne diseases as complex socio-ecological systems—a *‘people and nature’* paradigm—is needed to identify local interventions and transformative system-wide changes that could reduce human disease burden. We discuss longstanding scientific narratives of human involvement in zoonotic disease systems, which have largely framed people as ecological disruptors, and discuss three emerging research areas that provide wider system perspectives: how anthropogenic ecosystems construct new niches for infectious disease, feedbacks between disease, biodiversity and social vulnerability and the role of human-to-animal pathogen transmission (‘spillback’) in zoonotic disease systems. We conclude by discussing new opportunities to better understand the predictability of human disease outcomes from biodiversity change and to integrate ecological drivers of disease into health intervention design and evaluation.

This article is part of the discussion meeting issue ‘Bending the curve towards nature recovery: building on Georgina Mace's legacy for a biodiverse future’.

## 1. Introduction

Biodiversity and ecosystem processes underpin human health and wellbeing, from food and water security to helping maintain a stable climate. Understanding these varied contributions to health, and their potential erosion by anthropogenic stressors such as climate change and land use change, has therefore emerged as one of the most pressing themes in biodiversity science [1–3]. Directly measuring and attributing the contributions of biodiversity to health, and so communicating their value to policymakers, is a significant challenge owing to their complexity: biodiversity–health links are mostly indirect (e.g. pollination contributing to food security), occur at scales ranging from the microbial to the planetary, and are mediated by numerous social and environmental forces that vary over space and time [4]. Zoonotic and vector-borne infectious diseases (i.e. infections transmitted to humans from animals and/or by arthropod vectors) are unusual in being both an urgent concern for global public health and a relatively direct, visible and measurable link between human health and local ecosystem processes [5]. The past decade has seen a swift succession of infectious disease crises, from massive regional epidemics of dengue, chikungunya, Ebola and Lassa fever, to the worldwide spread of COVID-19, Zika and mpox. These have turned public awareness and global health policy attention towards how the biodiversity crisis and climate change may be impacting infectious disease trends and sparked significant discussions around how best to include nature-based interventions in emerging disease and pandemic risk governance [6–8]. This is, consequently, an important historical juncture during which insights from ecology and biodiversity sciences could significantly contribute to improving global public health.

**Figure 1.**
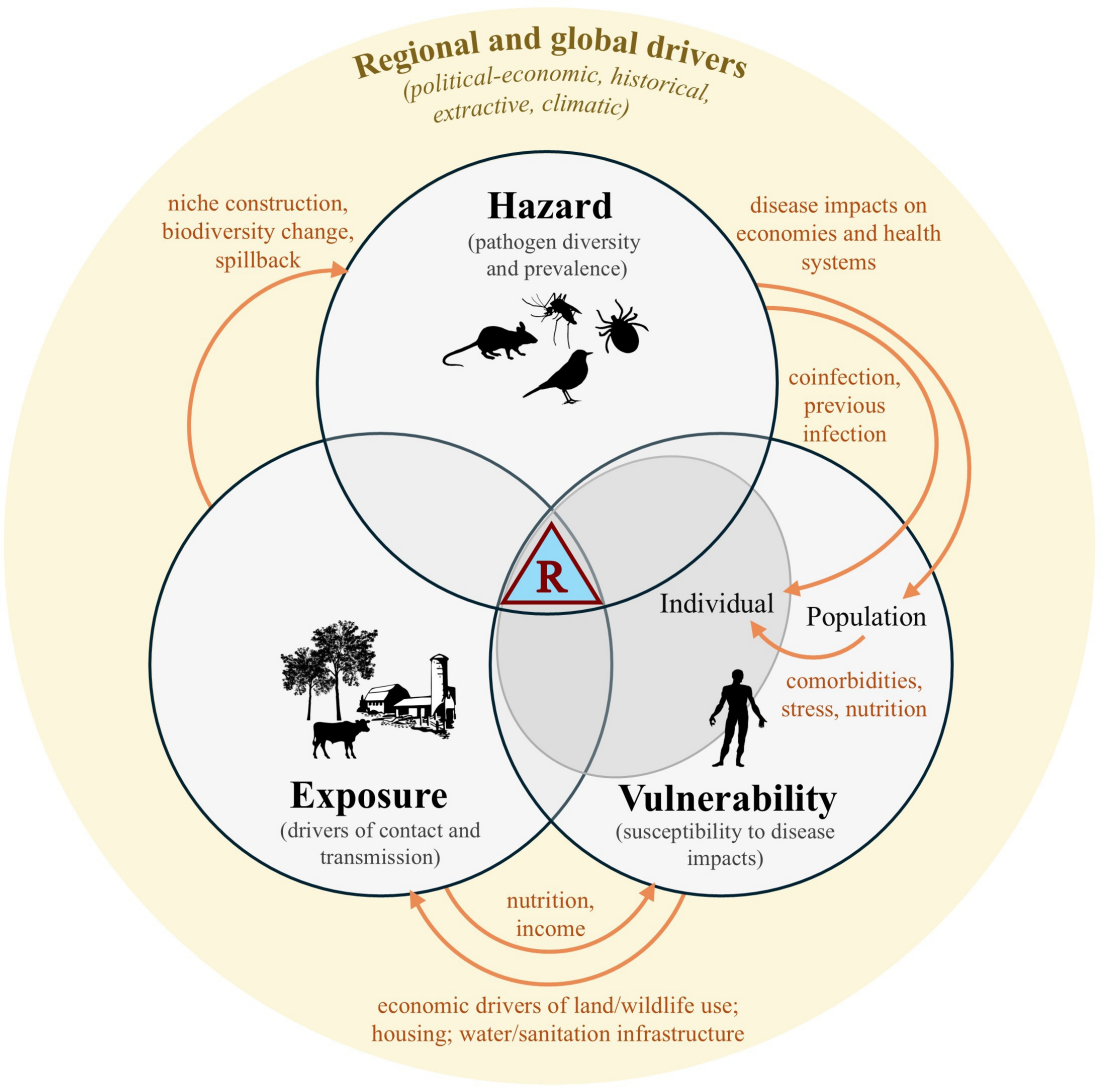
Local social–ecological feedbacks generate and reinforce infectious disease risks. Human zoonotic and vector-borne disease risks (blue triangle) arise from the convergence of circulating pathogen hazards within local ecosystems (shaped by host and pathogen diversity and prevalence), drivers that create opportunities for exposure (e.g. agricultural activities, urbanization and infrastructure, land conversion, wildlife hunting, hydrometeorological extremes) and individual- and population-level factors that influence vulnerability to disease and epidemics (e.g. individual physical condition and immunity; health systems access and functionality; inequality and social cohesion). Although often discussed and studied as separate phenomena [17], these processes are interdependent and subject to numerous feedback effects (orange arrows and text) that can generate or reinforce disease risks. For example, a high burden of disease (hazard) can impact community livelihoods and poverty (vulnerability), leading to increased reliance on local environment, land use or wildlife (exposure), which in turn can further impact local ecological communities (hazard). All these local processes are shaped by global forces, including unequal and extractive power relations, climate change and biodiversity degradation (yellow boundary), which can simultaneously impact pathogen hazards, exposure processes and vulnerabilities.

The emerging consensus in disease ecology is that anthropogenic ecosystem degradation and resulting ecological community changes—arising, for example, through habitat fragmentation, land use and climate change—on average tend to increase local pathogen transmission and disease in wildlife [5,9–11]. Long-term research in certain well-studied systems, such as Lyme disease in the US and Hendra virus disease in Australia, has demonstrated that these changes can have significant downstream impacts on infection risk to humans [12–14]. Yet the intervening role of social and socioeconomic processes in determining realized human disease outcomes remains poorly understood, particularly outside the high-income settings where biodiversity–disease relationships have been most intensely studied. Zoonotic and vector-borne disease systems are inherently socio-ecological in nature: modifications of landscapes for agriculture, industry and cities construct new niches and stressors for hosts, vectors and their pathogens, so shaping infection hazards [9,15]; social factors such as gender, wealth, livelihoods and nutrition influence human–wildlife contact and exposure to pathogens, susceptibility to disease and access to healthcare [16] (figure 1). What these processes look like varies widely across different regions and socioeconomic settings worldwide, and may depend more on political–economic and historical than proximate ecological circumstances [18–20]. Identifying policy strategies to improve the health of both people and ecosystems therefore requires us to understand not only the predictability of ecological drivers of disease hazards, but also how these are shaped, mitigated or amplified by human activities, and by social and economic processes. In this article, we review several major emerging themes in socio-ecological research into biodiversity and zoonotic and vector-borne disease risks. We outline the history of scientific perspectives on ecosystems and zoonotic infection, discuss three priority areas for research, and close by discussing opportunities to better integrate ecological drivers of disease into health intervention planning at various scales.

## 2. Towards a renewed ‘people and nature’ lens on biodiversity and disease

In an influential commentary published a decade ago, Georgina Mace offered a historical view of how scientific framings of biodiversity conservation have evolved over the past half-century, and their consequences for research, practice and policy [21]. She outlined how dominant paradigms within conservation science have historically been motivated either by protecting nature’s intrinsic value from threats posed by human activities (which she termed *‘nature despite people’*) or, more recently, maintaining the benefits and utility that people receive from ecosystems (i.e. ecosystem services; *‘nature for people’*). As many scholars have discussed, these long-standing scientific framings of the relationships between people, wildlife and ecosystems remain wedded to conservation’s roots in western philosophies, which position ‘nature’ as distinct and separate from human societies (and thus as an external resource open to exploitation) [22,23]. However, she also highlighted the more recent adoption of transdisciplinary conservation science framings that view humans as an inherent part of ecosystems, and which draw influence variously from socio-ecological systems theory, political ecology and Indigenous perspectives on nature [21]. Although still nascent in biodiversity sciences, such *‘people and nature’* perspectives —which emphasise the importance of socio-ecological interactions and feedbacks for maintaining resilient and healthy ecosystems—seem particularly well-suited to the challenges of understanding the complex ecologies of health and disease [24,25].

Socio-ecological systems approaches in biodiversity science share both intellectual roots and personnel with foundational early work on emerging infectious diseases, which described how pathogens emerge from the convergence of social, political-economic, eco-evolutionary and environmental forces that span scales from molecular to global [26–29]. Over a quarter-century later, this whole systems perspective persists in critical scholarship on disease emergence [19,30–32] and in the holism of organizing frameworks such as One Health and Planetary Health, which both frame human health as intrinsically dependent on the health of wildlife and ecosystems [33]. However, in practice, scientific knowledge production around emerging disease risks has stayed largely fragmented, discipline-specific and centred in Global North institutions. Epidemiological research has focused on proximate social risk factors and spread of disease in human populations, while rarely considering wider landscape and ecosystem contexts that shape infection and susceptibility. Conversely, disease ecology research has hugely advanced our understanding of multi-host infection dynamics in changing ecosystems, but while largely taking a simplified view of human involvement centred mainly on ecological disruption [32]. Dominant scientific and popular narratives tend to frame zoonotic spillover in *‘nature despite people’* conservation terms: human activities and behaviours produce risks by degrading biodiversity, encroaching into wild habitats or creating interfaces (e.g. expanding forest edges, live animal markets), across which pathogens make the ‘jump’ across the nature–society divide to threaten global health security. Humans are characterized as target hosts that receive (mostly) unidirectional flows of pathogens from risky wildlife host species [34,35], with zoonotic spillovers typically framed as rare and high-consequence events that might be preventable, including through conservation interventions. This kind of ecological outbreak narrative has substantially shaped wider public perceptions of zoonotic risks and influenced practice in research and policy, from local epidemiological investigation [36] to global advocacy around ecological levers for pandemic prevention [6].

Global health security-based framings of biodiversity and disease leave many important questions untouched, even as evidence points towards more complex realities. Only a small subset of known wildlife pathogens follow the pattern of sporadic spillovers leading to sustained global human-to-human epidemics or pandemics (such as SARS-CoVs, influenza A viruses and filoviruses) and, although ecological processes may contribute to index case spillover events, following emergence their overall burden and distribution depend on societal factors. In contrast, serological evidence for many zoonoses indicates that human infections are not rare and isolated events, but instead occur frequently in populations at risk, often during childhood, and do not always cause significant disease. This includes World Health Organization priority pathogens such as Lassa, Crimean–Congo haemorrhagic fever and Rift Valley fever viruses [37–39], indicating that many infections typically considered ‘emerging threats’ should instead be treated as neglected endemic diseases [40]. Indeed, a recent IUCN situation report concluded that most of the global human burden of zoonotic disease is endemic and attributable to recurring spillovers within anthropogenic habitats, transmitted by vectors, livestock and synanthropic wildlife [41]. Growing evidence also suggests that human-to-animal pathogen transmission (‘spillback’) might contribute substantially to pathogen maintenance and evolution in anthropogenic landscapes—a contrast to the general framing of ecosystems as sources of infection [42].

Together, this evidence indicates that cross-species transmission of microorganisms—among wildlife and livestock, to humans and from humans—is a relatively ubiquitous ecological process [43,44]. Understanding how biodiversity (and its loss) contribute to the burden of human infectious disease therefore requires a renewed attention to the social and ecological interactions that determine not only human infection risk (i.e. the spillover process) [45] but also the consequent impacts of disease on individuals and populations. This requires asking broader questions, such as: how do human-constructed habitats shape both short-term routes and dynamics of pathogen transmission, and the longer-term evolution of hosts, vectors and microorganisms? Under what circumstances, and for whom, are ecologically-driven changes in infection risk most consequential for health? What sets of interventions—ecosystem-based, health systems-based or otherwise—could be most effective at simultaneously curbing biodiversity loss and reducing the burden of disease? To explore these questions in more depth, the following sections discuss emerging research priorities in three areas: how human landscapes construct distinct niches for infectious disease; feedbacks between ecosystems and social vulnerabilities to infection; and the potential role of spillback in generating and sustaining disease risks.

## 3. Ecological communities and disease dynamics within anthropogenic ecosystems

**Figure 2.**
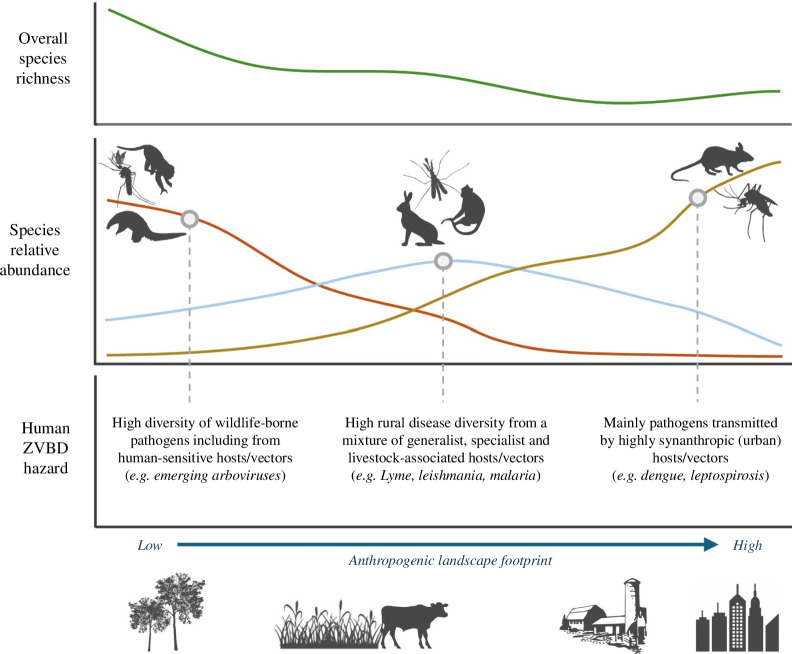
Compositional shifts in host and disease diversity under intensifying land use. Increasing intensities of anthropogenic land use intensity, from minimally disturbed habitat to rural ecosystems, towards high-intensity agriculture and urbanization, are generally associated with declines in overall local biodiversity (top graph). This overall trend masks compositional shifts in host and arthropod vector diversity that can significantly alter the local diversity and burden of zoonotic and vector-borne disease (ZVBD; middle and bottom graphs), including losses of more human-sensitive taxa (and their specialist pathogens; red line), the rise of highly synanthropic species (yellow line) and variable or hump-shaped responses of other species (blue line). These community shifts will reshape local disease transmission ecologies and create differences in disease diversity and burden in different types of landscape (examples in bottom graph captions). A deeper understanding of these changes in host, vector and pathogen diversity in response to human pressures at the level of biomes or ecoregions—for example, through leveraging global biodiversity data—may facilitate prediction of the infectious disease consequences of biodiversity change for many under-studied pathogens (see §6).

Since most pathogen exposures occur around homes or at work (e.g. via agricultural activities), zoonotic risks arise within environments that are often profoundly shaped by human activities [46]. Within these settings, invertebrate vectors such as mosquitoes and ticks play an important role in spreading infection either from animals to humans (e.g. borrelioses, rickettsial fevers, yellow fever, West Nile fever) or from human to human (e.g. dengue, chikungunya). High densities of livestock can support increased vector populations, act as bridging hosts for wildlife-borne infections and as key reservoirs for pathogen evolution and emergence (e.g. highly pathogenic avian influenzas in industrial poultry setups). Direct transmission from wildlife appears rarer—with certain exceptions, such as viruses transmitted by synanthropic rodents (e.g. arenaviruses and hantaviruses). The local taxonomic and functional diversity of host and vector communities thus determine the specific diversity, prevalence, transmission potential and evolution of pathogen hazards [9] (figure 2).

Human landscape modifications construct new configurations of infectious disease, both by creating new resource aggregations and ecological opportunities for resilient or invasive hosts and vectors to exploit, and by releasing remaining species from top–down ecological regulation by the predators and competitors that are extirpated [47,48]. On average, these community transitions tend to decrease biodiversity and favour increasing transmission and prevalence of multi-host pathogens in the remaining, more resilient host species (the dilution effect) [10,49]. However, the overall picture for human disease burden is likely complex, as the responses of different pathogens—many of which are maintained in complex multi-species sylvatic cycles—inherently depend on the varied responses of their host and vector assemblages. For example, increasing land use intensity tends to favour rodent and bat zoonotic hosts and certain competent *Culex* and *Aedes* mosquito vectors of human disease, while other groups such as primates and certain *Anopheles* mosquitoes decline [34,50,51]. Rather than directional trends in total disease incidence, the end result may often be compositional shifts in pathogen diversity, and thus a transition in disease syndromes and burden in human populations (figure 2). For example, in Brazil, increasing anthropogenic landscape transformation correlates with a transition away from parasitic diseases transmitted by rural vectors (malaria, leishmaniasis) and towards urban mosquito-transmitted arboviral diseases (dengue, Zika, chikungunya) [52]. Importantly, those host species that persist in close proximity to humans tend to be more stressed, which can impact immunocompetence, pathogen prevalence and shedding, and so exacerbate human risks (reviewed in [53]). A key emerging theme for disease ecology is therefore to better understand how infection dynamics are shaped by the distinct ecologies and climates of anthropogenic landscapes, and how this might differ across regional syndromes, modes and intensities of land use [44].

Understanding and predicting how host and pathogen communities vary across anthropogenic landscapes requires a deeper knowledge of why certain species can tolerate or thrive in close proximity to people (*synanthropy*). At the species level, species trait syndromes that are associated with resilience to anthropogenic pressures (e.g. fast life histories, wide geographic ranges) also tend to correlate to observed zoonotic reservoir status [54,55]—meaning that (known) zoonotic hosts disproportionately persist in human-disturbed landscapes [34,56]. However, increasing evidence indicates that synanthropy is not entirely species-intrinsic, but can be region-specific, historically contingent and plastic in response to the opportunities—and stresses—of particular human-driven environments. Such differences can significantly impact spatial interaction networks and foci of infection risk. For example, the space-use of macaques in fragmented forest–agricultural landscapes in Borneo influences their prevalence of zoonotic malaria infection (*Plasmodium knowlesi*), and micro-hotspots of human exposure emerge at forest edges where interactions between macaques, mosquito vectors and people are highest [57]; conversely, macaque *P. knowlesi* prevalence is often lower in peri-urban habitat, likely owing to the absence of suitable vectors [58]. The social inequalities that structure urban communities, environments and infrastructure—for example, historical district redlining in US cities [59]—can strongly impact local species diversity and composition, with poorly understood implications for pathogen exposure. At a broader scale, the multimammate rat *Mastomys natalensis* shows significant variation in synanthropy between its East and West African subpopulations, living in closest proximity to human homes in rural West Africa—where it acts as principal Lassa fever reservoir—but rarely occurring in urban areas owing to competitive exclusion by invasive rodent species [60]. Over longer timescales, adaptations to urban living can lead to evolutionary transitions to synanthropy [61], such as the divergence of human-specialist *Aedes aegypti* mosquitoes from their tree hole-breeding ancestor several hundred years ago [62], or recent morphological changes in urbanizing UK urban fox populations [63].

Compared to minimally disturbed ecosystems, anthropogenic habitats often show markedly different—and in many cases more extreme—environmental variability over time, such as seasonal fluctuations in water availability (e.g. transient versus year-round), vegetation and food resources (e.g. cropping cycles) and local climates (e.g. heat island effects). Many zoonotic and vector-borne diseases are climate sensitive, with temperature, rainfall and humidity impacting host and vector population dynamics, physiology and body condition, behaviour and host–pathogen interactions, as well as pathogen persistence in the external environment [64]. Theory and a burgeoning empirical literature show that the synergistic effects of land use and climate can therefore significantly modify the timing, intensity and spatial patterns of spillover and epidemics. Such impacts may often be mediated by wildlife host populations and behaviour, such as the combined impacts of El Niño droughts and fragmented habitat in driving Hendra virus spillover from fruit bats [13], or oscillations in rodent host populations and viral infection in agro-ecosystems where seasonal cropping determines food availability [65]. However, climate–land use–disease synergies have so far been more commonly studied for vector-borne infections [66]. For example, there is evidence that impacts of temperature on malaria transmission in Venezuela are more pronounced in gold mining areas [67], and that dengue incidence increases more sharply after extreme rainfall in rural than urban areas of Brazil (potentially because better urban drainage infrastructure reduces vector breeding sites) [68]. Accounting for these kinds of interactions will be crucial to improve outbreak forecasting and preparedness in a changing climate [69]. Importantly, as these examples indicate, human-driven landscapes and their amplifying or mitigating effects on climate-sensitive diseases are not homogeneous, but are instead shaped by historical and spatial socioeconomic disparities [70]. In densely built-up and poorer neighbourhoods, urban heat island effects and low-quality water and sanitation infrastructure can increase both thermal suitability and breeding site availability for mosquito reproduction and vector competence, and thereby concentrate arbovirus burden in marginalized communities [69,71]. As we discuss below, such interactions at the nexus of ecology, economy, climate and infection have potential to further reinforce and embed existing social vulnerabilities.

## 4. How does the socio-ecological context shape exposure and susceptibility to infection?

To date, research into relationships between ecosystem change and human infectious disease has principally focused on changing assemblages of hosts, vectors and pathogens (i.e. shifts in hazards, as described above; figure 1) [17]. Focusing too narrowly on this dimension, however, risks missing the bigger picture of how ecological degradation contributes to how people are differentially exposed and made vulnerable to infection [26,28]. Social inequalities across dimensions such as gender, race, wealth and livelihood, and ensuing biosocial determinants such as nutrition, coinfection, stress and immunocompetence, shape who is most susceptible to disease, who gets sick following infection and whether disease is then diagnosed, treated and reported [28]. The interplay of ecological and biosocial processes in driving infectious disease outcomes is complex (figure 1) and still poorly understood. Degradation of ecosystem processes can influence not only circulating pathogen diversity but also human immunocompetence and susceptibility to disease, for example by impacting nutrition, water security and coinfection. Poverty and social marginalization increase exposure to many of these pathogens while simultaneously reducing access to healthcare, reinforcing disparities in the health and economic burden of disease, for example between richer and poorer communities (figure 1). More ecologically degraded areas are also less resilient to climate extremes and disasters such as flooding and landslides [72], which affect infrastructure, livelihoods and infection risks and potentially further increase pressure on local biodiversity. Theoretical models indicate that feedbacks like these can have serious societal implications, for example suggesting the existence of self-sustaining rural ‘poverty traps’, driven by vicious cycles of disease and economic burden [73,74]. The statistical challenge of disentangling such complex relationships from observational data means that empirical examples remain sparse, with some notable exceptions: for example, there is evidence for negative malaria–deforestation feedbacks in the Brazilian Amazon [75].

In some circumstances, the combined effects of ecosystem degradation, climate change and socioeconomic inequalities can simultaneously increase both people’s exposure to (multiple) wildlife-borne pathogens and, once infected, their susceptibility to disease. Such interactions between multiple pathologies are studied in public health as *syndemics* (synergistic epidemics), i.e. situations where the co-occurrence of more than one health or social condition produces worse outcomes than either in isolation, with a canonical human example being substance misuse, violence and HIV/AIDS [76–78]. Biodiversity-disease research has largely focused on hazards within single-pathogen single-disease systems, so shared drivers and probable syndemic interactions with wider social, ecological and health conditions—including inequalities, comorbidities and coinfection—are still poorly understood [79–81]. Studying these interactions has clear importance for public health, for example by helping to understand why many important zoonotic and vector-borne infections show such wide variation in clinical severity. Social and ecological circumstances shape individuals’ lifetime pathogen exposure histories and thereby their susceptibility to subsequent infections, which could either increase (e.g. heterotypic dengue virus infection [82]) or decrease the risk of severe disease (e.g. early-life microbial exposures potentially priming the immune system against future infections [83,84]). Closely overlapping niches across different pathogens—arising via shared host and vector communities—can also cluster coinfection risks in space, time and in response to ecological drivers, increasing the potential for complex multi-pathogen interactions [44]. For example, *Aedes*-borne arboviruses (e.g. dengue, Zika, chikungunya) and water-borne infections (e.g. leptospirosis) tend to cluster in poorer and peripheral neighbourhoods of tropical cities [85]. Coinfection can worsen or complicate individual disease outcomes [80], but co-circulation at population level can also worsen outcomes by interfering with prompt diagnosis, especially for rarer and non-specific infections. In West Africa, initial misdiagnosis of Lassa fever as malaria can delay appropriate treatment by several days, increasing the risk of mortality [86]. Historical and ongoing ecosystem degradation and social marginalization, for example in racialized and Indigenous communities, also impact rates of non-communicable disease in ways that exacerbate susceptibility to many infections [87]; one particularly visible recent example was the clustering of COVID-19 morbidities and mortality in many marginalized communities [88]. One so-far-neglected role for ecologists in supporting public health decisions would be better understanding and mapping of such compound hazards, their intersection with other health and economic conditions and, crucially, the upstream forces (including neocolonial relationships with Global North states and financial interests; figure 1) that generate and sustain them [18,19].

These socio-ecological complexities present both opportunities and challenges for designing and evaluating interventions to reduce disease risks. Social and behavioural change interventions can be designed to target human activities that increase risk of direct and indirect forms of animal contact (e.g. wildlife hunting; use of animal products as fertilizer; food storage practices); these should aim to increase knowledge, risk perception or self-efficacy, and take care not to stigmatize cultural and need-based practices [89]. Ecological interventions could be designed to reduce sylvatic circulation and human hazards for groups of priority pathogens at regional levels—for example, via conservation, restoration or agroecosystem management activities [24,90]. Yet diseases differ widely in their hosts, pathogen life cycles and socio-ecological contexts, indicating that effective one-size-fits-all ecological interventions may be rare [44]. Certain global solutions might even risk unintended consequences for other dimensions of health if applied too broadly. For example, tighter regulation of deforestation and wildlife trade have been proposed as global solutions to mitigate emerging zoonosis risks [6]. However, the militarised turn in conservation [91] and examples of land dispossessions under schemes such as REDD+ [92] indicate that the way that such programmes are implemented could have significant health consequences for local and Indigenous communities, with potential to be unjust and counterproductive; these may include loss of land, nutrition and income sources, stress and exposure to other infections. More positively, multiple social and ecological system ‘weak points’ could be targeted to variously interrupt transmission, reduce susceptibility to disease and improve healthcare access, and the most effective strategies for net health benefits may consider several at once. For example, hunting of wild meat is an important transmission pathway for multiple zoonoses, but also important for food, nutrition and economic security [93–95]; any disease risk interventions targeting this pathway would need to take these interactions (and possible unintended consequences of regulation) into account (figure 1). The early and sustained involvement of affected communities is therefore critical to ensuring the design of socio-ecological interventions that are equitable, recognize and value local knowledge and perspectives, and thereby provide net benefits to health and wellbeing (see §6).

## 5. The role of human-to-animal pathogen transmission in sustaining zoonotic risks

Scientific narratives around disease emergence have traditionally centred on humans as target hosts of spillover infections from wildlife reservoirs. This anthropocentric view has been shaped, in large part, by ongoing (and understandable) research biases towards hosts and pathogens of known medical relevance to humans [96]. However, continual improvements in genomic/metagenomic tools and network science methods now offer an increasingly broad, holistic view of a continuous flow of microorganisms among humans, non-humans and their environments [43,97]. One important implication of this shift in perspective is that people and livestock are, in fact, increasingly large and well-connected nodes in multi-species pathogen sharing networks, and so may play substantial roles in transmitting infection to other species (i.e. zooanthroponosis or ‘spillback’) [98]. Pathogen transmission from people to wildlife poses a well-known threat to certain wildlife populations, such as respiratory virus spillback to some primates [42]. In contrast, much remains unknown about how human-to-wildlife or livestock-to-wildlife transmission—particularly to synanthropic species with which we share landscapes—might be important in generating and sustaining zoonotic risks.

The consequences of spillback can include the establishment of reservoirs within new hosts and geographic locations: for example, multiple introductions of SARS-CoV-2 have established endemic circulation in white-tailed deer in North America, which could ultimately serve as a source of novel variants to re-emerge in humans [99]. Human-to-wildlife transmission might also contribute to maintaining long-term (enzootic) pathogen prevalence in highly dynamic synanthropic host populations, such as rodent hosts of arenaviruses [100], but its significance remains poorly understood. Livestock populations can also play important epidemiological roles, including by sustaining epizootics, supporting arthropod vector populations and acting as conduits for infection between disparate geographical areas; for example, cattle may play a significant (but still poorly defined) role in the ecology of Crimean–Congo haemorrhagic fever [101]. Genomic tools offer a promising route to identify these probable sources and sinks for infection in multi-host systems [102]; one recent such study at the global scale suggests that spillback may be very widespread, although this pattern is difficult to distinguish conclusively from human-centric surveillance biases at this scale [98]. Moving forward, more granular datasets that are not solely focused on wildlife hosts or vectors—for example, samples and individual-level metadata collected from human, wildlife and livestock populations in the same landscapes [97]—will afford opportunities to test hypotheses around the relative frequency, drivers and epidemiological significance of spillback in human-driven landscapes.

## 6. Future horizons

Over the past two decades, research into biodiversity and infectious disease has largely been motivated by two parallel missions—to reduce biodiversity loss and ecosystem degradation and to improve human health—and the tensions and trade-offs between these two desired outcomes have not always been as clearly articulated as their possible synergies. Recent progress towards a reconciled scientific consensus on biodiversity–disease relationships has helped to defuse longstanding debates in disease ecology and provided important nuance [5,49,103]. Yet we remain far from an actionable science that could, for example, provide well-grounded predictions of how specific land use or conservation policies would affect the net burden of human disease (and its wider social and economic ramifications). As we have discussed, this issue arises from both the inherent challenge of measuring the many contributions of biodiversity to human health, and the need to better understand the social settings that drive pathogen transmission and disease (figure 1). Nonetheless, patterns of infectious disease are underpinned by general ecological and evolutionary processes that should provide sources of predictability that can inform policy, even across diverse social contexts. In the later stages of her career Georgina Mace focused on bridging communication gaps between research and policy; in that spirit, this final section offers a reflection on emerging opportunities to implement biodiversity–disease knowledge to improve health.

Looking forward, a crucial step in any disease system is to identify the most effective intervention strategies that could aim to reduce hazards, exposure, vulnerability, or potentially a combination of all three (figure 1). More targeted activities could address proximal drivers of specific diseases, focusing variously on lowering pathogen prevalence within host populations (e.g. land use policies, ecological restoration, host or vector control, wildlife vaccination), interrupting human exposure pathways (e.g. reducing hunting or trade of high-risk species, improving water and sanitation infrastructure), reducing human susceptibility to disease (e.g. vaccination, improving food security, better prevention and treatment of comorbidities) or ensuring prompt diagnosis and treatment following infection (e.g. by improving health systems infrastructure and accessibility). Improvements in statistical methods for causal inference from observational data are increasingly entering ecology from the health sciences [104]. Applied to infection datasets from people and wildlife, these offer a promising means to infer likely drivers of disease risk (e.g. climate, land use, poverty, etc.), which can then inform the iterative design and evaluation of combined ecological, social and/or health system interventions. Large-scale, long-term human disease surveillance datasets are crucial to robustly attribute climatic drivers [105,106]; however, these datasets suffer from pervasive geographical surveillance biases that make detecting more localized, transient, lagged or synergistic driver effects—such as local land use changes—far more challenging [44]. Unpacking how ecosystem-based interventions could reduce disease will often instead require starting locally. Simultaneous surveys of people, wildlife, livestock and environments can enable the identification of key pathways and drivers of cross-species infection; when combined with emerging genomic and network science methods, these have potential to significantly improve our understanding of pathogen diversity and sharing across space, time and species.

These natural sciences-based approaches can provide a starting point by identifying critical risk pathways to target, but achieving truly effective and just interventions will require breaking with the dominant paradigm of exploitative, top-down and parachute research. Early and ongoing involvement of affected communities in co-design and implementation are key to ensuring that proposed interventions are rooted in local knowledge, perspectives and existing resilience or risk-mitigation strategies. Participatory studies and intervention scenario mapping with at-risk communities, as well as integrating local representatives into research teams and accountability structures, can help in achieving this [107]. Ensuring sustainable net benefits to local health and wellbeing will likely involve accounting for numerous ecosystem services beyond just disease risk, as well as addressing social disparities such as healthcare access. Recent and ongoing work on zoonotic malaria and Lassa fever has shown the value of such detailed socio-ecological research for informing disease prediction and prevention [57,108]. Shifting research norms towards participatory and co-design approaches will require tackling significant institutional and structural barriers. These include improving academic institutional and funding support for interdisciplinary research and the development of equitable North–South research partnerships, recognising that these relationships are slower to establish and require the development of trust, shared values and working frameworks across significant geographical and economic distances, and so may often be slower to produce measurable outputs. They also include the need for institutional commitments and funding to close persistent capacity gaps between Global North and Global South research communities that sustain inequities in research and knowledge, including (but not limited to) building analytical, data science and evidence synthesis capacities, and disincentivizing parachute research.

In parallel with local-scale interventions, there is also an obvious need to provide better recommendations around biodiversity impacts on disease to regional and global health and environmental policymakers. This need has been clearly demonstrated in the formation of the United Nations’ cross-sectoral One Health Quadripartite collaboration and One Health High-Level Expert Panel [8], and recent discussions around the priority of ecosystem-based ‘primary prevention’ activities in global emerging disease governance [6,7]. At these broader scales, the limitations and biases inherent to pathogen surveillance data for many emerging infections become more visible and problematic [44]. However, it also becomes possible to leverage data from wider biodiversity and environmental monitoring initiatives to help identify the ecological changes that underpin disease risks – from host community responses to environmental pressures [34,109], to geographies of viral diversity and their eco-evolutionary histories [110], to regional syndromes of anthropogenic landscape and socioeconomic pressures and their political-economic drivers [111,112]. Achieving this potential will require facilitating discussions between biodiversity monitoring, disease ecology and disease surveillance communities to align on data and metadata standards, ensure relevant data are collected and reported wherever possible, and ensure proper source attribution (for example, Verena’s PHAROS database standard; https://pharos.viralemergence.org/). An important future analytical challenge will then be to identify at what spatial scales such macro-level monitoring data could be translated to useful information for disease prediction and intervention activities. For example, host, vector and pathogen communities may show relatively consistent and predictable responses to land use and climate change within individual ecoregions or regional biomes [113,114] (figure 2). In many cases, it may be possible to align these with similarly region-specific typologies of land change, agroecology, priority social and health issues, and policy stakeholder networks, where such data exist and are accessible [111]. Integrating the disease costs of ecosystem change into health economic analyses—including the distinct economic burdens of endemic and epidemic infections—may also be most tractable and policy-relevant at these regional or national scales [115]. There are clear synergies, then, between enhancing biodiversity monitoring and strengthening health systems-based disease detection, treatment and response: both have potential to significantly improve our baseline knowledge and situational awareness, which will be especially valuable in the context of rapid global change.

Finally, ‘people and nature’ perspectives on wildlife-borne disease offer a reminder to pose questions that, as Richard Levins wrote, are big enough to encompass the problems we seek to address [26,116]. As we have discussed, much of the research into biodiversity and disease dynamics over the past two decades has made important advances via the study of specific wildlife disease systems, proximal rather than upstream drivers, and development of ecological theory. Yet the COVID-19 pandemic highlighted the current limits of this ecological knowledge to either inform specific health and environmental decisions, or contextualize disease emergence as a product of the wider, interlinked systemic crises of capital, inequality, biodiversity and climate change [28,31] (figure 1). Looking forward, socio-ecological approaches to biodiversity and infectious disease research have great potential to contribute to this much wider understanding, and so help to identify and advocate for transformative system-wide interventions—including expanding global access to high-quality healthcare, climate change mitigation, economic redistribution and challenging powerful financial interests—that will support sustainable, just and healthy futures for ecosystems and people.

## Data Availability

This article has no additional data.
